# A Combined Effect of Polygenic Scores and Environmental Factors on Individual Differences in Depression Level

**DOI:** 10.3390/genes14071355

**Published:** 2023-06-27

**Authors:** Anastasiya Kazantseva, Yuliya Davydova, Renata Enikeeva, Rustam Mustafin, Sergey Malykh, Marina Lobaskova, Alexander Kanapin, Inga Prokopenko, Elza Khusnutdinova

**Affiliations:** 1 Institute of Biochemistry and Genetics—Subdivision of the Ufa Federal Research Centre of the Russian Academy of Sciences, 450054 Ufa, Russia; julia.dmitrievna@list.ru (Y.D.); enikeevarf@gmail.com (R.E.); elzakh@mail.ru (E.K.); 2 Laboratory of Neurocognitive Genomics, Department of Genetics and Fundamental Medicine, Ufa University of Science and Technology, 450076 Ufa, Russia; a.kanapin@gmail.com; 3 Department of Medical Genetics and Fundamental Medicine, Bashkir State Medical University, 450008 Ufa, Russia; ruji79@mail.ru; 4 Psychological Institute, Russian Academy of Education, 125009 Moscow, Russia; malykhsb@mail.ru (S.M.); lobaskovamm@mail.ru (M.L.); 5 Department of Psychology, Lomonosov Moscow State University, 125009 Moscow, Russia; 6 Department of Clinical & Experimental Medicine, University of Surrey, Guildford GU2 7XH, UK; i.prokopenko@surrey.ac.uk; 7 People-Centred Artificial Intelligence Institute, University of Surrey, Guildford GU2 7XH, UK

**Keywords:** depression, polygenic score, social/lifestyle factors, regression models, sensitivity analysis

## Abstract

The risk of depression could be evaluated through its multifactorial nature using the polygenic score (PGS) approach. Assuming a “clinical continuum” hypothesis of mental diseases, a preliminary assessment of individuals with elevated risk for developing depression in a non-clinical group is of high relevance. In turn, epidemiological studies suggest including social/lifestyle factors together with PGS to address the “missing heritability” problem. We designed regression models, which included PGS using 27 SNPs and social/lifestyle factors to explain individual differences in depression levels in high-education students from the Volga–Ural region (VUR) of Eurasia. Since issues related to population stratification in PGS scores may lead to imprecise variant effect estimates, we aimed to examine a sensitivity of PGS calculated on summary statistics of depression and neuroticism GWAS from Western Europeans to assess individual proneness to depression levels in the examined sample of Eastern Europeans. A depression score was assessed using the revised version of the Beck Depression Inventory (BDI) in 1065 young adults (age 18–25 years, 79% women, Eastern European ancestry). The models based on weighted PGS demonstrated higher sensitivity to evaluate depression level in the full dataset, explaining up to 2.4% of the variance (*p* = 3.42 × 10^−7^); the addition of social parameters enhanced the strength of the model (adjusted r^2^ = 15%, *p* < 2.2 × 10^−16^). A higher effect was observed in models based on weighted PGS in the women group, explaining up to 3.9% (*p* = 6.03 × 10^−9^) of variance in depression level assuming a combined SNPs effect and 17% (*p* < 2.2 × 10^−16^)—with the addition of social factors in the model. We failed to estimate BDI-measured depression based on summary statistics from Western Europeans GWAS of clinical depression. Although regression models based on PGS from neuroticism (depression-related trait) GWAS in Europeans were associated with a depression level in our sample (adjusted r^2^ = 0.43%, *p* = 0.019—for unweighted model), the effect was mainly attributed to the inclusion of social/lifestyle factors as predictors in these models (adjusted r^2^ = 15%, *p* < 2.2 × 10^−16^—for unweighted model). In conclusion, constructed PGS models contribute to a proportion of interindividual variability in BDI-measured depression in high-education students, especially women, from the VUR of Eurasia. External factors, including the specificity of rearing in childhood, used as predictors, improve the predictive ability of these models. Implementation of ethnicity-specific effect estimates in such modeling is important for individual risk assessment.

## 1. Introduction

Depression is a well-established cause of disability worldwide, which affects 4.4% of the world population [1]. Based on psychogenetics research, a heritability coefficient of depression accounts for 35–50% [2], thus promoting a plethora of molecular-genetic studies. Multiple efforts were made to unravel the hypothesis-driven [3,4,5] and/or hypothesis-free approaches [6,7,8,9,10,11] to dissect a genetic cause of depression. Existing molecular mechanisms underlying depressive pathology are based on the impaired neurotransmitter signaling [12], hypothalamic–pituitary–adrenal (HPA) axis [5], oxytocin and arginine vasopressin systems [4,13,14], inflammatory response [3,15], telomere length-associated molecular pathways [16,17], nucleotide changes in target mRNA-miRNA binding sites [18,19], etc. Genome-wide association studies (GWAS) of depression and their meta-analyses implementing a hypothesis-free approach identified multiple genetic loci [7,8,9,11,20,21,22]. Although many of GWAS’ significant SNPs appear to reside in intergenic regions, their regulatory role in associated molecular pathways has to be clarified.

Single genetic variants (even highly significant at the GWAS level) only confer a small effect on disease manifestation, thus limiting our ability to evaluate individual disease susceptibility. Therefore, simultaneous estimation of SNP effects may help to address the “missing heritability” problem. A polygenic score (PGS) approach, as one of the techniques aimed to estimate a simultaneous effect of multiple genetic loci on a phenotype of interest, became a widely used instrument during the past decade. To date, several GWAS-based PGS studies of clinical depression (diagnosed with major depressive disorder, MDD) have been published, demonstrating the ability of such models to assess liability to develop depression and related comorbid disorders [23,24]. Together with PGS estimating a risk for complex phenotypes based on GWAS estimates [25,26], certain studies sought to examine PGS implementing a limited number of attributed genetic variants based on their functional relevance to molecular mechanisms of a disease [27,28,29]. However, a proportion of variance in liability to depression explained by a combined genetic impact varies from 0.5 to 3% [26,28].

In turn, various environmental factors can trigger the development of clinical depression symptoms in susceptible individuals. The impact of environmental factors has been examined via epidemiological studies and those implicating gene-by-environment interaction approaches. The last ones have been conducted in terms of SNP association studies [30,31] and PGS-by-environment effects [32]. According to our previous research [4] and existing epidemiological data, such factors as childhood trauma [30], stressful life events, child–parent relationships, parenting behavior [33,34], socioeconomic status, and family income [35] can significantly contribute to depression liability, partially due to the epigenetic reprogramming of HPA axis signaling [36]. In turn, epidemiological studies suggest the inclusion of demographic/lifestyle factors in the statistical model together with PGS to address the “missing heritability” problem. However, to date, the number of studies ascertaining an integrative effect of genetic scores and social variables on liability to depression under the PGS paradigm is insufficient. Moreover, they are mainly focused on a limited number of examined social factors, such as body mass index (BMI) [32], childhood maltreatment [24,37], and smoking behavior [38]. Therefore, research examining a combined effect of various social predictors and PGS in statistical models will help to increase the proportion of variance explained in liability to depression.

Assuming a “clinical continuum” hypothesis of mental diseases, it is of high importance to design PGS models to assess individual susceptibility or proneness to depression, even in a mentally healthy population cohort. In addition, depression as a multifactorial mental disorder is known to manifest under certain circumstances, thus providing a rationale for estimating genetically-mediated depression levels in younger adults. However, existing studies incorporating the PGS approach to evaluate differences in depressive-like traits in a general cohort are mainly based on GWAS of the clinical depression [23,25,26,27]. Nevertheless, attempts to detect associations between expression-based polygenic risk scores and depressive symptoms in a non-clinical cohort have been made [39]. Implementing the PGS approach, to date, several attempts have been made to examine PGS from clinical depression to assess anxiety- and depression-related traits in non-clinical cohorts of the European ancestry [23,25,26]. Moreover, state-of-the-art research, including the phenome-wide approaches, confirmed a significant burden of depression co-morbidity with a broad range of diseases, i.e., MDD PGS was significantly associated with an enhanced risk for developing 22 various complex phenotypes, including anxiety and sleep disorders [23]. These studies provide a rationale to examine if PGS models based on summary statistics from clinical depression can also explain individual differences in depression levels in a general cohort.

To date, a plethora of PGS studies of depression and related phenotypes are mainly based on Western Europeans, including UK Biobank cohort [23,25,26]. However, issues related to population stratification in PGS estimates may lead to imprecise variant effect estimates for genetic scores if those are transferred directly from other populations. Previous studies indicated that PGS based on scores revealed by UK Biobank was inappropriate to correctly classify an individual’s liability for developing depression in the East Asian ancestry [37]. This demands a higher number of PGS studies on depression involving non-European and Eastern European individuals. In this regard, the possibility of using genetic scores calculated on the basis of GWAS-derived data from Western European populations to evaluate depression levels in other populations of Eastern European ancestry (for instance, Russian-descent individuals) has to be addressed.

In the present study, we aimed to use the PGS approach based on effect estimates from 27 SNPs examined in the Volga–Ural region of Eurasia and social/lifestyle factors to explain the individual differences in BDI-measured depression in the higher-education students. For sensitivity purposes, we aimed to estimate if PGS based on summary statistics of depression and neuroticism GWAS in Western Europeans could assess individual proneness to depression levels in the examined sample of Eastern Europeans.

## 2. Materials and Methods

### 2.1. Participants

Overall, we included 1065 higher-education students from the Volga–Ural region of Eurasia (DeprVUR; mean age ± SD: 19.53 ± 1.75 years; age range: 18–25 years; 79% women). All the respondents were students at Universities in Russia of European ancestry (357 Russians, 340 Tatars, 234 Udmurts, and 134 individuals of mixed ethnicity). Individuals with a self-reported individual history of any mental disorder and suicidal thoughts and actions in the past were excluded. Enrolled volunteers were asked to respond to the questionnaire on sociodemographic parameters, including sex, age, and specificity of rearing in childhood (rearing in a complete/incomplete family, family income, and maltreatment).

The study was approved by the Biological Ethics Committee at the Institute of Biochemistry and Genetics—Subdivision of the Ufa Federal Research Centre of the Russian Academy of Sciences (Ufa, Russia) (protocol code 15, date of approval, 12 October 2017). Written informed consent was obtained from all participants after they were acquainted with the procedures. All participants were informed about the voluntary and confidential nature of their participation. All procedures performed were in accordance with the ethical standards of the institutional and/or national research committee and with the 1964 Helsinki Declaration and its later amendments or comparable ethical standards.

### 2.2. Psychological Assessment

We used the revised Russian version of the self-report Beck Depression Inventory (BDI-II) for the assessment of depression level [40]. It is a 21-item multiple-choice questionnaire to quantitatively measure depression severity on the basis of cognitive–affective and somatic depression subscales.

The Parental Bonding Instrument (PBI, 25 items) [41] was used to evaluate maternal and paternal styles of parenting based on two bipolar scales (“care” and “protection”). The PBI recalls child-rearing attitudes separately toward maternal and paternal styles, which are assessed on a four-point Likert scale. A summed number of “care” items positively correlates with a degree of parental warmth toward offspring during childhood. A “protection” score reflects the perceptions of how parents controlled their child’s decision-making. The assignment to “high” or “low” categories was based on the following cut-off scores: a “care” score of 27.0 and a “protection” score of 13.5 for mothers; a “care” score of 24.0 and a “protection” score of 12.5 for fathers.

### 2.3. Blood Sample Collection, SNPs Selection, and Genotyping

We obtained peripheral blood samples from each participant in 8 mL EDTA-containing vacutainer tubes. Subsequently, genomic DNA was isolated from blood leukocytes via the phenol–chloroform extraction technique. DNA concentration was measured with a NanoDrop 1000 spectrophotometer (Thermo Fisher Scientific, Fitchburg, WI, USA) and used at a final concentration of 30 ng/μL.

We selected 32 SNPs (Supplementary Table S1) from the dbSNP database with a minor allele frequency (MAF) higher than 0.05 in Europeans (according to the 1000 Genomes Project, accessed on 10 April 2023), based on their association with depression and affective pathology in previous GWAS of Western Europeans. Selected SNPs were enriched in the genes, which ablation or enhanced production of encoding proteins resulted in depression-related phenotype in animal studies, including genes belonging to hypothalamic–pituitary–adrenal, monoaminergic, inflammatory response, and miRNA binding pathways. The *OXTR* rs13316193 (*p* = 2.15 × 10^−11^) and *HTR2A* rs7322347 (*p* = 0.0031) deviated from the Hardy–Weinberg equilibrium (HWE); therefore, they were excluded from the subsequent analysis.

Genotyping of selected SNPs was carried out with a competitive allele-specific PCR (KASP) technology (LGC Genomics, Aarhus, Denmark) using CFX96 Touch™ Real-Time PCR Detection System (BioRad, Hercules, CA, USA). DNA samples were amplified in a total volume of 10 μL, 0.14 μL KASP Assay mix, and 5 μL KASP-TF Master Mix (LGC Genomics, Denmark). Alleles were assigned based on fluorescence end-point analysis with the CFX Manager™ Software (BioRad, USA). All SNPs demonstrated sufficient call rates (>98%).

### 2.4. Statistical Analysis

To calculate the genotype and allele frequencies of all examined SNPs and to manage the HWE test, we used PLINK v.1.9 [42]. A measure of linkage disequilibrium between SNPs located in the same genetic locus was assessed via r^2^ (PLINK v.1.9). The Kolmogorov–Smirnov’s test was used (SPSS v.23, SPSS Inc., Chicago, IL, USA) to examine the correspondence of BDI-measured depression score to the normality of distribution. The effect of social–demographic categorical variables on individual variance in depression level was evaluated via the Mann–Whitney U test (SPSS v.23). For this analysis, we applied a correction for multiple testing for the number of examined social–demographic parameters (P_FDR_ < 0.05/8), which provided us with a threshold of P_FDR_ < 0.0063.

To estimate the main effects of genetic variants on depression levels and to obtain standardized regression coefficients for each SNP, a series of linear regression analyses was carried out adjusted for sex, age, and ethnicity in the total sample under the additive effect of SNPs in PLINK v.1.9. Obtained regression coefficients served as effect estimates for the subsequent examination of the association of individual polygenic scores and depression level in young adults under linear regression in R v.4.1.2 [43].

### 2.5. PGS Calculation and Regression Models

The PGS calculation was carried out for each subject from the DeprVUR sample as a weighted sum of all examined genetic variants based on the following formula:

PGS = β_1_x_1_ + β_2_x_2_ +…+ β_n_x_n,_ (1)

where β_i_—effect estimate (standardized regression coefficient) for ith SNP; x_i_—individual dosage of the effect allele (number of effect alleles).

The calculation of PGS for each individual was performed using PLINK v.1.9; the number of effect alleles at each locus was multiplied by the standardized regression coefficient (β). These coefficients have been initially obtained under the additive linear regression model adjusted by sex, age, and ethnicity in the sample from the Volga–Ural region of Eurasia. In the cases of negative β, we used an opposite sign of regression coefficient, which was multiplied by the number of alternative alleles in that case, to obtain the association with an enhanced depression score.

To provide the required assumptions for PGS calculation, we excluded three SNPs due to the existence of proxy SNPs among the analyzed list of SNPs. Namely, the *OXTR* rs237911—with rs2228485 (r^2^ = 0.44); the *TNF* rs1041981—with rs1800629 (r^2^ = 0.23); the *FKBP5* rs1360780—with rs3800373 (r^2^ = 0.55) in the examined DeprVUR sample. Therefore, PGS-based models were constructed on the basis of 27 SNPs.

To examine the cumulative impact of genetic variants (PGS) and social–demographic variables on depression level and to assess the percent of the variance in depression explained by predictors, we analyzed four different linear regression models, including the following predictors: (1) PGS; (2) sex, ethnicity, and age; (3) PGS, sex, age, and ethnicity; (4) PGS, age, sex, ethnicity, and the most significant social predictors. A set of the most significant lifestyle/social predictors in Model 4 was established using a stepwise backward elimination function in R [43], based on the best values of Akaike information criterion, effect size, and *p*-values. Adjusted r^2^ (determination coefficient) described a proportion of variance in BDI-measured depression for all examined models. For sensitivity purposes, we assessed the effect of unweighted PGS in the total sample and weighted PGS in women. Due to a small number of males (*n* = 224), we did not perform sensitivity analysis in men. Unweighted PGS was calculated as the sum of effect alleles (in the case of positive β) and alternative alleles (in the case of negative β) based on initial linear regression analysis.

Since, to date, no summary statistics have been available for the selected SNPs on depression-like phenotype in the Russian cohort, we performed a search for GWAS studies of depression and anxiety-related traits with available summary statistics, which have been conducted in individuals of European ancestry (Supplementary Table S2). As a result of our search, we identified well-powered GWAS studies of depression [7,9,10] and neuroticism (anxiety-related trait) [7,20]. Therefore, we calculated weighted and unweighted PGS for each individual in the DeprVUR sample based on summary statistics from mentioned studies for sensitivity purposes.

## 3. Results

### 3.1. Phenotypic Characteristics of the Study Sample

The characteristics of the study sample (DeprVUR) are reported in Table 1. Almost all of the analyzed sociodemographic factors significantly affected individual differences in depression levels after FDR correction. Namely, sex (*p* = 0.0041), income level (*p* = 0.0031), maltreatment in childhood (*p* = 0.0033), and parental style of rearing (*p* < 0.001) contributed to the variance in depression levels in young adults. To be more precise, an enhanced depression was more prominent in women, individuals with lower than average income level, who reported childhood maltreatment, low levels of parental care, and increased parental protection. This observation points to a rationale for the inclusion of significant social/lifestyle factors as predictors together with PGS in statistical models.

### 3.2. Association Analysis

A set of 31 examined SNPs was included in the present study after quality control checks. Allele frequencies and their effects on depression levels in the DeprVUR sample are shown in Table 2. As a result of linear regression analysis conducted in the DeprVUR sample controlling for sex, ethnicity, and age, we observed significant effects of the *PCLO* rs2715157 A-allele (β = 0.67, *p* = 0.03) and the *IL18* rs187238 C-allele (β = 0.73, *p* = 0.03) on BDI-measured depression.

### 3.3. Effect of Weighted PGS and Social/Lifestyle Factors on BDI-Measured Depression Based on DeprVUR Estimates

**Figure 1:**
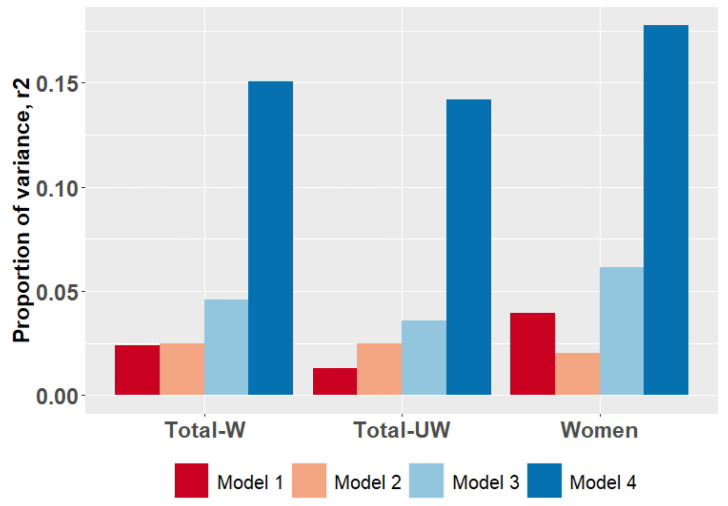
Proportion of variance (adjusted r^2^) in BDI-measured depression explained by predictors in DeprVUR sample based on weighted (W) PGS score. For sensitivity analysis, unweighted (UW) PGS effects in DeprVUR sample and weighted PGS in women were examined. In all groups, examined predictors in four different linear regression models were as follows: (1) PGS; (2) sex, ethnicity, and age; (3) PGS, sex, age, and ethnicity; (4) PGS, age, sex, ethnicity, and the most significant social/lifestyle predictors. Included predictors are described in detail in Table 3.

While performing weighted PGS analysis, we observed that a cumulative effect of SNPs (Model 1) accounted for 2.4% of the variance in depression score in the DeprVUR sample (p_model_ = 3.42 × 10^−7^). The effect of sex, ethnicity, and age (Model 2) on depression levels was also evident (p_model_ = 1.21 × 10^−5^); it explains up to 2.5% of the variance in the examined phenotype. A combined effect of SNPs, sex, age, and ethnicity on depression (Model 3) was more pronounced and explained up to 4.6% of the variance (p_model_ = 1.15 × 10^−9^). The final examined regression model (Model 4), which represented a combined effect of genetic variants and lifestyle factors on depression levels, explained up to 15% of the variance (p_model_ < 2.2 × 10^−16^) (Table 3, Figure 1). Based on a stepwise backward elimination algorithm, almost all of the examined social/lifestyle factors except for maltreatment and rearing in a complete/incomplete family significantly contributed to Model 4. In general, individuals with higher PGS, being women of younger age, characterized by low-income childhoods, low parental care, and overprotection, appeared to be more prone to have higher depression levels.

### 3.4. PGS-Based Sensitivity Analysis Based on DeprVUR Estimates

In the second stage, we examined whether the unweighted PGSs were sensitive to explain the depression level in DeprVUR of the total sample. We observed that the effects of unweighted PGS including models (p_model_ = 1.61 × 10^−4^, r^2^ = 0.013 for Model 1; p_model_ = 1.49 × 10^−7^, r^2^ = 0.036 for Model 3; p_model_ = < 2.2 × 10^−16^, r^2^ = 0.14 for Model 4) were less significant than of including weighted PGS (Table S3, Figure 1).

In addition, we examined the sensitivity of weighted PGS to assess depression liability in women due to a high prevalence in our cohort. Model 1, depicting the effect of SNPs, was sensitive to estimating higher depression levels in women (p_model_ = 6.03 × 10^−9^) and explained up to 3.9% of the variance (Table S3, Figure 1). The addition of sex, ethnicity, age (Model 3, p_model_ = 1.17 × 10^−10^, r^2^ = 0.061), and social factors (Model 4, p_model_ < 2.2 × 10^−16^, r^2^ = 0.17) into the model together with PGS enhanced a prognostic value of regression model.

### 3.5. PGS-Based Sensitivity Analysis Based on GWAS Estimates in Europeans

To address the question on the applicability of effect estimates obtained from GWAS of depression and related phenotypes in Western European populations for evaluating depression levels in Eastern Europeans (for instance, Russian-descent cohort), we calculated both weighted and unweighted PGS for each individual from the DeprVUR sample. For this purpose, we obtained the effect estimates from the summary statistics of publicly available GWAS data of the unipolar depression [7,9,10]. However, PGS based on clinical depression effect estimates failed to significantly explain higher BDI-measured depression in the DeprVUR sample (*p* > 0.05 for weighted and unweighted PGS, data are available on request). Subsequently, we examined PGS models based on the summary statistics from neuroticism (depression-related trait) GWAS in the Europeans [7,20] to assess BDI-measured depression in individuals from the Volga–Ural region. Although we observed a significant effect of Models 2 and 3 on explaining depression in the DeprVUR sample controlling social/lifestyle predictors, it seemed that the impact of genetic variants (Model 1) was rather small and significant only in the case of unweighted PGS effect (p_model_ = 0.019 [7], p_model_ = 0.043 [20]) (Table S4, Figure S1). Moreover, unweighted PGS explained a higher proportion of variance in depression compared to weighted PGS (r^2^ = 0.0043 vs. r^2^ = 0.0027—for Okbay et al. [7]; r^2^ = 0.029 vs. r^2^ = 0.0021—for Turley et al. [20]).

The inclusion of sex, age, ethnicity, and social factors as predictors improved the model’s prediction sensitivity (p_model_ < 2.2 × 10^−16^ for Model 4; r^2^ = 0.14 and 0.15 for weighted and unweighted effects based on [7]; r^2^ = 0.15 for weighted and unweighted effects based on [20]) (Table S4, Figure S1).

## 4. Discussion

In this study, for the first time, we assessed the ability to explain individual differences in depression levels in higher-education students from the Volga–Ural region of Eurasia, implementing a polygenic score approach. The designed PGS model was based on 27 SNPs residing in the genes belonging to hypothalamic–pituitary–adrenal, monoaminergic, inflammatory response, and miRNA binding pathways. This PGS model explained up to 2.4% of the variance in depression score in the DeprVUR sample, while the addition of sex, age, and ethnicity as predictors to the model together with PGS increases the proportion of variance up to 4.6%. To date, several studies examining a cumulative contribution of a limited number of genetic variants to depression liability have been reported in European [32] and non-European populations [37]. We identified suggestive evidence of PGS-including models to evaluate depression. Similar to our findings, PGS of depression based either on a limited number of SNPs [37] or on a complete list of GWAS-available genetic variants [26] explained ~2–3% of depressive symptoms. It should be noted that PGS was based on genetic variants, which have been previously associated with impaired mental health/psychopathologies in GWAS and simultaneously related to molecular pathways involved in depression development. However, the demonstrated effect of PGS to evaluate individual liability to depressive personality in the DeprVUR sample is probably attributed to a significant impact of the *PCLO* rs2715157 and the *IL18* rs187238 (*p* < 0.05) on depression score. The *PCLO* rs2715157 has been primarily established in MDD GWAS in Europeans [8], while *IL18* rs187238 was associated with clinical depression [3], and a differential expression level of *IL18* gene was related to rs187238 genotypes [44].

In the present study, we determined the regression models based on both PGS and social parameters, which explained up to 15% (total DeprVUR sample) and 17% (sensitivity analysis in women) of individual variance in depression levels. These values were attributed to the effect of social predictors, including specificity of rearing in childhood, at a larger extent than to a cumulative impact of SNPs (2.4% in the total DeprVUR sample and 3.9% in women). Previous studies also indicated a modulatory role of external factors, especially early life adverse events, on depression manifestation. For instance, our previous research demonstrates a significant effect of parental rearing style on *OXTR* gene-related association with negative personality in the examined cohort [45]. In line with our findings, several PGS-based studies estimated a combined effect of PGS and non-genetic factors to evaluate depression liability, including such classical demographic variables as sex and age [32], childhood adversity [37], smoking behavior, and number of alcoholic drinks per week [38]. Notably, PGS based either on a limited number of SNPs [32,37] as in our study or on a complete list of GWAS-available SNPs [26] explained ~2–3% of the variance in depressive symptoms, while the addition of environmental factors (even classic demographic variables, such as age, sex, and ethnicity) improved a prediction ability of PGS-including model. For instance, the percent of the variance in depressive symptoms in healthy adolescents and young adults explained by GWAS-based PGS of depression [46] varied depending on responders’ age from 0.37% in 10-year-olds to 2.21% in 23-year-olds [26]. In summary, published data on a smaller proportion of phenotypic variance related to a cumulative impact of SNPs compared to even classic demographic variables have been confirmed by our research group. However, the explained variance can be increased in some cases by a simultaneous analysis of a larger number of SNPs as was reported, for instance, for sleep quality (PGS based on 5000 SNPs accounted for ~9%) [47].

As a result of sensitivity analysis, constructed PGS-based model significantly explained variability in BDI-measured depression separately in women, even to a greater extent. Namely, a cumulative effect of SNPs and the addition of sex, age, and ethnicity as predictors explained up to 3.9% and 6.1% of the variance in women compared to 2.4% and 4.6% in the total sample, respectively. This result can be explained by a greater proportion of women and by significantly higher mean depression level in women than in men in the DeprVUR sample, which coincides with a widely established two-fold increase in the risk of depression and related traits in women [2].

Despite multiple studies indicating the possibility of using PGS based on effect estimates from related phenotypes to estimate one another, the number of PGS studies based on effect estimates from clinical depression (i.e., MDD, unipolar depression) to evaluate individual proneness to non-clinical depressive states remain scarce. For example, Guffanti et al. [25] examined whether PGS was based on Okbay et al.’s [7] depression-related phenotypes can explain individual variance in anhedonia-related traits in mentally healthy volunteers. The authors reported a significant negative association of genetic scores on the changes in striatal reward prediction induced by stress, while no PGS effect was observed on self-reported pleasure after exposure to psychosocial stressors [25]. Another study also succeeded in predicting worse depressive symptoms in the sample of healthy adolescents and young adults based on neuroticism and clinical depression PGS [26]. In turn, the phenome-wide study of MDD PGS reported significant prediction ability for developing 22 various complex phenotypes, including anxiety [23]. Within a framework of sensitivity analysis, we failed to explain variability in depression levels in a non-clinical cohort of young adults based on summary statistics from depression GWAS. Similar to our findings, Pearson-Fuhrhop et al. [27] detected that PGS based on clinical depression-related estimates from the discovery sample insignificantly explained individual variance in depression levels in mentally healthy individuals. Moreover, a large-scale study conducted in the Swedish population evidenced that family genetic risk scores for depression, bipolar disorder, and schizophrenia could clearly separate affective disorders from psychotic ones [48], thus evidencing a unique polygenic score profile of a certain mental disorder or a psychological profile.

Another finding coming from the analysis performed by our research group demonstrates insensitivity of neuroticism PGS based on summary statistics from Western Europeans [7,20] to explain the variance in depression levels in subjects from the Volga–Ural region of Eurasia. This conclusion is based on our findings that unweighted neuroticism-based PGS of Western Europeans demonstrated a more significant effect on evaluating BDI-measured depression in the DeprVUR sample than weighted PGS. Such failure can be attributed to significant interethnic differences in genotype frequencies, which have to be considered while transmitting genetic data obtained from one population to another. These results point to the necessity to construct PGS based on the effect estimates from GWAS of the same or ethnically close population.

It should be noted that the present research has several prompts, including a relatively homogenous cohort of young adults by age and level of education (they were all students at the universities). We also included a number of social/lifestyle factors, including child–parent relations, as predictors. However, the reported findings have several limitations. First, PGS was calculated on the basis of a limited number of examined genetic variants, thus resulting in a small value of the proportion of variance explained by PGS. It should also be mentioned that to date, GWAS of depression conducted in a cohort of Russian descent was carried out [49]; however, summary statistics from this study remain publicly unavailable. Therefore, for the sensitivity analysis, we calculated PGS based on effect estimates from GWAS of depression/neuroticism of Western Europeans. Moreover, the discovery sample of higher-education-attaining young adults had a moderate sample size; thus, observed findings have to be verified in a larger replication sample of same-age individuals from the same geographic location. Finally, the findings obtained are potentially biased by the overrepresentation of women in the examined sample, thus indicating higher sensitivity to explain individual variance in depression levels in women from the VUR.

## 5. Conclusions

In summary, the present study is a preliminary attempt to construct models implementing the PGS approach to explain individual liability for manifesting depressive states under the age of 25 years in individuals from the Volga–Ural region of Eurasia. The weighted model considering PGS and social/lifestyle factors as predictors demonstrated the best prognostic ability and accounted for up to 15% of the variance in depression score in the DeprVUR sample and 17% in the women group. However, a combined effect of selected SNPs explained up to 2.4% and 3.9% of the variance in depression levels in the DeprVUR sample and among women, respectively. This observation indicates a significant impact of social factors, including specificity of rearing in childhood, in individual differences in depression.

Nevertheless, our data points to a weak prognostic ability of models implementing the PGS approach calculated on the basis of summary statistics obtained from neuroticism and Depression GWAS of other ethnic groups (i.e., Western Europeans). Naturally, future research in this field would benefit from the use of the PGS approach based on GWAS summary statistics of depression or related phenotypes conducted in Eastern Europeans, including the Volga–Ural region of Eurasia. On the other side, summary statistics from the whole genome, transcriptome, metabolome, and other “omics” data can become useful for calculating the PGS-based individual liability to depression.

## Figures and Tables

**Table 1:** Characteristics of DeprVUR sample (*n* = 1065) and the effect of social/lifestyle factors on BDI-measured depression.

Parameter	*n* (%)	Mean Depression Score ± SD	*p*-Value
Sex			**0.0041**
Men	221 (20.75)	7.50 ± 7.32	
Women	844 (79.25)	8.59 ± 7.05	
Family income			**0.0031**
lower than average	113 (10.57)	10.67 ± 8.59	
average and higher	952 (89.43)	8.04 ± 6.94	
Rearing in full family			0.891
yes	892 (83.72)	8.33 ± 7.13	
no	173 (16.28)	8.19 ± 6.84	
Maltreatment			**0.0033**
yes	104 (9.78)	10.67 ± 8.43	
no	961 (90.22)	7.99 ± 7.03	
Maternal care			**<0.001**
high	728 (68.33)	7.07 ± 6.05	
low	337 (31.67)	11.19 ± 8.73	
Maternal protection			**<0.001**
high	575 (4.02)	9.74 ± 7.85	
low	490 (45.98)	6.76 ± 6.13	
Paternal care			**<0.001**
high	564 (52.95)	7.07 ± 6.44	
low	501 (47.05)	9.70 ± 7.83	
Paternal protection			**<0.001**
high	500 (46.91)	9.63 ± 8.25	
low	565 (53.09)	7.14 ± 6.00	

Abbreviations: SD—standard deviation. Statistically significant *p*-values after correction for multiple comparisons are marked in bold.

**Table 2:** Effects of 31 examined genetic variants on BDI-Depression in DeprVUR sample (*n* = 1065).

SNP	Gene	Chr:Position (GRCh38)	EA/NEA	EAF	β	SE	*p*-Value	Direction of Effect
rs3093077	*CRP*	1:159709846	G/T	0.079	−0.24	0.58	0.665	-
rs33911258	*AVPR1B*	1:206118034	G/A	0.167	0.01	0.42	0.990	-
rs1800587	*IL1A*	2:112785383	A/G	0.278	0.29	0.35	0.397	-
rs16944	*IL1B*	2:112837290	A/G	0.378	−0.26	0.32	0.411	-
rs7632287	*OXTR*	3:8749760	A/G	0.199	−0.16	0.38	0.661	-
rs2254298	*OXTR*	3:8760542	A/G	0.095	−0.13	0.54	0.795	-
rs53576	*OXTR*	3:8762685	A/G	0.473	−0.04	0.31	0.874	+
rs2228485	*OXTR*	3:8768017	G/A	0.208	−0.25	0.38	0.493	+
rs237911 *	*OXTR*	3:8768322	G/A	0.165	−0.04	0.42	0.928	*NA*
rs9818870	*MRAS*	3:138403280	T/C	0.139	0.36	0.46	0.424	-
rs1317082	*TERC*	3:169779797	G/A	0.341	−0.01	0.33	0.987	-
rs7726159	*TERT*	5:1282204	A/C	0.337	−0.31	0.33	0.339	-
rs41423247	*NR3C1*	5:143399010	C/G	0.352	−0.04	0.33	0.898	+
rs1800629	*TNF*	6:31575254	A/G	0.109	0.04	0.51	0.931	-
rs1041981 *	*TNF*	6:31573007	A/C	0.248	−0.46	0.35	0.193	*NA*
rs3800373	*FKBP5*	6:35574699	C/A	0.234	−0.01	0.36	0.978	-
rs1360780 *	*FKBP5*	6:35639794	T/C	0.280	−0.11	0.35	0.752	-
rs13212041	*HTR1B*	6:77461407	C/T	0.177	0.12	0.40	0.754	+
rs10457441	*MIR2113*	6:98124244	C/T	0.419	0.48	0.32	0.128	-
rs2148710	*FYN*	6:111801023	T/C	0.135	0.32	0.45	0.480	+
rs2715157	*PCLO*	7:82839058	A/G	0.438	0.67	0.31	**0.031**	*NA*
rs531564	*MIR124*	8:3445535	C/G	0.151	−0.22	0.44	0.610	-
rs2487999	*OBFC1*	10:103900068	T/C	0.087	0.65	0.56	0.240	+
rs1800955	*DRD4*	11:636784	C/T	0.402	−0.26	0.31	0.390	-
rs187238	*IL18*	11:112164265	G/C	0.283	−0.73	0.35	**0.034**	-
rs3803107	*AVPR1A*	12:63147054	T/C	0.176	0.64	0.41	0.120	-
rs1042615	*AVPR1A*	12:63150429	A/G	0.403	−0.03	0.31	0.920	+
rs10459194	*MIR135*	12:99039512	C/T	0.302	0.52	0.34	0.125	-
rs2230912	*P2RX7*	12:121184393	G/A	0.170	−0.01	0.41	0.995	+
rs1042173	*SLC6A4*	17:30197993	T/G	0.454	0.32	0.32	0.303	-

Abbreviations: EA/NEA—effect (minor) allele/non-effect (major) allele; EAF—effect allele frequency; β—regression coefficient. Statistically significant allele effects are shown in bold. Sex, ethnicity, and age are included in linear regression models as covariates. Proxy SNPs in the *OXTR*, *TNF*, and *FKBP5* genes, which have been excluded from the PGS calculation after LD checks, are marked with an asterisk. Direction of effect is shown relative to effect estimates from Neuroticism GWAS [7]. NA - data non-available.

**Table 3:** Linear regression models demonstrating the effect of SNP-based PGS and social/lifestyle predictors on BDI-measured Depression in DeprVUR sample (*n* = 1065).

Model	Parameter	β	SE	*p*-Value
1	PGS	57.65	11.23	3.42 × 10^−7^
	Model *p*-value	3.42 × 10^−7^		
	Adjusted r^2^	0.024		
2	Sex	1.12	0.54	0.038
	Ethnicity (Russians)	−1.34	0.76	0.079
	Ethnicity (Tatars)	−2.77	0.76	2.96 × 10^−4^
	Ethnicity (Udmurts)	−1.95	0.83	0.019
	Age	−0.30	0.13	0.022
	Model *p*-value	1.21 × 10^−5^		
	Adjusted r^2^	0.025		
3	PGS	53.44	11.19	2.05 × 10^−6^
	Sex	1.07	0.54	0.045
	Ethnicity (Russians)	−1.60	0.76	0.034
	Ethnicity (Tatars)	−2.74	0.76	2.95 × 10^−4^
	Ethnicity (Udmurts)	−1.89	0.83	0.021
	Age	−0.30	0.13	0.018
	Model *p*-value	1.15 × 10^−9^		
	Adjusted r^2^	0.046		
4	PGS	52.70	13.27	7.98 × 10^−5^
	Sex	1.50	0.63	0.017
	Ethnicity (Russians)	−1.68	0.88	0.055
	Ethnicity (Tatars)	−2.54	0.84	0.0026
	Ethnicity (Udmurts)	−1.34	1.01	0.18
	Age	−0.27	0.14	0.056
	Income level (average)	−1.90	0.87	0.028
	Maternal care	−2.65	0.59	9.29 × 10^−6^
	Maternal protection	1.52	0.56	0.0063
	Paternal care	−1.07	0.54	0.047
	Paternal protection	1.43	0.55	0.0091
	Model *p*-value	<2.2 × 10^−16^		
	Adjusted r^2^	0.15		

The best social/lifestyle predictors according to stepwise backward elimination procedure are included in Model 4.

## Data Availability

Not applicable.

## Supplementary Materials

The following supporting information can be downloaded at: https://www.mdpi.com/article/10.3390/genes14071355/s1, Figure S1: Proportion of variance (adjusted r^2^) in BDI-measured depression explained by predictors in DeprVUR sample based on weighted (W) and unweighted (UW) PGS score [7,20]; Table S1: Effects of examined SNPs on risk of depression and other mental diseases in PheWAS (FinnGen5) and GWAS catalog [8,50,51,52,53,54,55,56,57,58,59,60]; Table S2: Effects of examined genetic variants on BDI-Depression in DeprVUR (*n* = 1065) and on neuroticism in previous GWAS; Table S3: Linear regression models demonstrating weighted and unweighted PGS effect on BDI-measured Depression in DeprVUR and women group (for sensitivity purposes); Table S4: Sensitivity models demonstrating the effect of PGS (controlling for various covariates) based on published GWAS data of neuroticism on BDI-measured Depression in DeprVUR sample.
