# Supplementary material for: A Combined Effect of Polygenic Scores and Environmental Factors on Individual Differences in Depression Level
Source: Genes (Basel). 2023 Jun 27;14(7):1355. doi: 10.3390/genes14071355 (PMC10379734; doi:10.3390/genes14071355)
Supplement: Supplementary file 1 [file genes-14-01355-s001.zip › genes-2403937-supplementary.docx]

Supplementary Tables

**Table S1.** Effects of examined SNPs on risk of depression and other mental diseases in PheWAS (FinnGen5) and GWAS catalogue [1-12].

| **SNP** | **Gene Name** | **Chr:position**  **(GRCh38)** | **EA/NEA** | **Associated phenotype** | **P-value** | **Cases/controls** | **Reference** |
| --- | --- | --- | --- | --- | --- | --- | --- |
| rs3093077 | *CRP* | 1: 159709846 | G/T | [Neurotic disorders](https://r5.finngen.fi/pheno/F5_NEUROTICOTH)  ADHD | 2.5x10^-2^  2.0x10^-6^ | 991 / 198,110  22406 | FinnGen5  [1] |
| rs33911258 | *AVPR1B* | 1: 206118034 | G/A | [Somatoform disorder](https://r5.finngen.fi/pheno/F5_SOMATOFORM) | 3.6x10^-2^ | 2,627 / 198,110 | FinnGen5 |
| rs1800587 | *IL1A* | 2: 112785383 | A/G | [Schizotypal disorder](https://r5.finngen.fi/pheno/F5_SCHIZOTYP)  Short-term memory | 1.0x10^-4^  7.0x10^-7^ | 668 / 208,674  738 | FinnGen5  [2] |
| rs16944 | *IL1B* | 2: 112837290 | A/G | [Sleep disorders](https://r5.finngen.fi/pheno/F5_SLEEP)  [Conduct disorder](https://r5.finngen.fi/pheno/F5_CONDUCT) | 1.5x10^-3^  1.0x10^-2^ | 332 / 216,164  407 / 215,763 | FinnGen5 |
| rs7632287 | *OXTR* | 3: 8749760 | A/G | [Disorder of the sleep-wake schedule](https://r5.finngen.fi/pheno/F5_SLEEPWAKE) | 2.0x10^-3^ | 190 / 216,164 | FinnGen5 |
| rs2254298 | *OXTR* | 3: 8760542 | A/G | [Anxiety disorders](https://r5.finngen.fi/pheno/KRA_PSY_ANXIETY) | 1.5x10^-3^ | 20,992 / 197,800 | FinnGen5 |
| rs53576 | *OXTR* | 3: 8762685 | A/G | Mental and behavioural disorders | 5.1x10^-2^ | 571 / 214,999 | FinnGen5 |
| rs2228485 | *OXTR* | 3: 8768017 | G/A | [Anxiety disorders](https://r5.finngen.fi/pheno/KRA_PSY_ANXIETY_EXMORE)  Depression | 2.9x10^-2^  2.0x10^-6^ | 20,992 / 166,584  874 | FinnGen5  [3] |
| rs237911 | *OXTR* | 3: 8768322 | G/A | [Disorder of the sleep-wake schedule](https://r5.finngen.fi/pheno/F5_SLEEPWAKE) | 8.3x10^-3^ | 190 / 216,164 | FinnGen5 |
| rs13316193 | *OXTR* | 3 : 8761057 | C/T | [Disorder of the sleep-wake schedule](https://r5.finngen.fi/pheno/F5_SLEEPWAKE) | 5.0x10^-3^ | 190 / 216,164 | FinnGen5 |
| rs9818870 | *MRAS* | 3: 138403280 | T/C | [Substance abuse](https://r5.finngen.fi/pheno/KRA_PSY_SUBSTANCE_EXMORE) | 2.0x10^-2^ | 12,819 / 166,549 | FinnGen5 |
| rs1317082 | *TERC* | 3: 169779797 | G/A | [Insomnia](https://r5.finngen.fi/pheno/F5_INSOMNIA) | 3.0x10^-2^ | 1,691 / 216,164 | FinnGen5 |
| rs7726159 | *TERT* | 5: 1282204 | A/C | [Anankastic personality disorder](https://r5.finngen.fi/pheno/F5_ANAPER) | 1.5x10^-2^ | 476 / 212,179 | FinnGen5 |
| rs41423247 | *NR3C1* | 5: 143399010 | C/G | [Persistent mood disorders](https://r5.finngen.fi/pheno/F5_PERSMOOD)  [All anxiety disorders](https://r5.finngen.fi/pheno/F5_ALLANXIOUS)  Cognitive traits | 3.2x10^-2^  4.2x10^-2^  4.0x10^-6^ | 3,901 / 192,220  12,513 / 19,811  13,500 | FinnGen5  FinnGen5  [4] |
| rs1800629 | *TNF* | 6: 31575254 | A/G | [Bipolar affective disorders](https://r5.finngen.fi/pheno/F5_BIPO)  Educational attainment | 8.0x10^-3^  4.0x10^-9^ | 4,501 / 192,220  510,795 | FinnGen5  [5] |
| rs1041981 | *TNF* | 6: 31573007 | A/C | [Bipolar affective disorders](https://r5.finngen.fi/pheno/F5_BIPO) | 1.8x10^-2^ | 4,501 / 192,220 | FinnGen5 |
| rs3800373 | *FKBP5* | 6: 35574699 | C/A | [Neurotic, stress-related and somatoform disorders](https://r5.finngen.fi/pheno/F5_NEUROTIC)  [Anxiety disorders](https://r5.finngen.fi/pheno/KRA_PSY_ANXIETY) | 7.1x10^-3^  1.0x10^-2^ | 20,682 / 198,110  20,992 / 197,800 | FinnGen5 |
| rs1360780 | *FKBP5* | 6: 35639794 | T/C | [Reaction to severe stress](https://r5.finngen.fi/pheno/F5_STRESSOTH)  [Anxiety disorders](https://r5.finngen.fi/pheno/KRA_PSY_ANXIETY) | 1.9x10^-2^  2.8x10^-2^ | 7,296 / 198,110  20,992 / 19,780 | FinnGen5 |
| rs13212041 | *HTR1B* | 6: 77461407 | C/T | [Depression](https://r5.finngen.fi/pheno/F5_DEPRESSIO) Educational attainment | 2.1x10^-2^  4x10^-12^ | 23,424 / 192,220  1131,438 | FinnGen5  [6] |
| rs10457441 | *MIR2113* | 6: 98124244 | C/T | [Bipolar affective disorders](https://r5.finngen.fi/pheno/F5_BIPO)  Neuroticism | 2.7x10^-4^  8.0x10^-12^ | 4,501 / 192,220  449,484 | FinnGen5  [7] |
| rs2148710 | *FYN* | 6: 111801023 | T/C | Aggressive behavior  [Schizoaffective disorder](https://r5.finngen.fi/pheno/F5_SCHIZOAFF) | 3.0x10^-8^  2.1x10^-2^ | 8,747  2,288 / 208,674 | [8]  FinnGen5 |
| rs2715157 | *PCLO* | 7: 82839058 | A/G | Unipolar depression | 3.0x10^-8^ | 1,942 / 4,565 | [9] |
| rs531564 | *MIR124* | 8: 9903189 | C/G | [Depression](https://r5.finngen.fi/pheno/F5_DEPRESSIO)  Neuroticism | 7.4x10^-4^  3.0x10^-21^ | 23,424 / 192,220  329,821 | FinnGen5  [10] |
| rs2487999 | *OBFC1* | 10: 103900068 | T/C | [Anxiety disorders](https://r5.finngen.fi/pheno/KRA_PSY_ANXIETY_EXMORE) | 3.9x10^-2^ | 20,992 / 166,584 | FinnGen5 |
| rs1800955 | *DRD4* | 11: 636784 | C/T | [Emotional disorders starting during childhood or adolescense](https://r5.finngen.fi/pheno/KRA_PSY_CHILDEMOT) | 3.4x10^-3^ | 805 / 217,987 | FinnGen5 |
| rs187238 | *IL18* | 11: 112164265 | G/C | [Depression](https://r5.finngen.fi/pheno/F5_DEPRESSIO) | 1.5x10^-2^ | 23,424 / 192,220 | FinnGen5 |
| rs1042615 | *AVPR1A* | 12: 63150429 | A/G | [Anankastic personality disorder](https://r5.finngen.fi/pheno/F5_ANAPER) | 1.2x10^-2^ | 476 / 212,179 | FinnGen5 |
| rs10459194 | *MIR135* | 12: 99039512 | C/T | [Emotional disorders](https://r5.finngen.fi/pheno/F5_EMOCHILD)  Bipolar disorder | 1.2x10^-5^  5.0x10^-9^ | 887 / 215,763  51,710 | FinnGen5  [11] |
| rs2230912 | *P2RX7R* | 12: 121184393 | G/A | [Somatoform disorder](https://r5.finngen.fi/pheno/F5_SOMATOFORM)  Schizophrenia | 6.5x10^-3^  3.0x10^-8^ | 2,627 / 198,110  77,096 | FinnGen5  [12] |
| rs7322347 | *HTR2A* | 13:46835968 | T/A | Conduct and emotional disorders | 2.0x10^-3^ | 416 / 218,376 | FinnGen5 |
| rs1042173 | *SLC6A4* | 17: 30197993 | T/G | [Bipolar affective disorders](https://r5.finngen.fi/pheno/F5_BIPO) | 7.1x10^-3^ | 4,501 / 192,220 | FinnGen5 |

Abbreviations: EA/NEA – effect allele/non-effect allele.

**Table S2.** Effects of examined genetic variants on BDI-Depression in DeprVUR (N=1,065) and on neuroticism in previous GWAS.

| **SNP** | **Gene** | **Chr:position**  **(GRCh38)** | **EA/NEA** | **DeprVUR**  **N=1,065** | | | | **Okbay et al., 2016** [13]  **N=170,911** | | | | **Turley et al., 2018** [14]  **N=168,105** | | | |
| --- | --- | --- | --- | --- | --- | --- | --- | --- | --- | --- | --- | --- | --- | --- | --- |
|  |  |  |  | **EAF** | **β** | **SE** | ***p*-value** | **EAF** | **β** | **SE** | ***p*-value** | **EAF** | **β** | **SE** | ***p*-value** |
| rs3093077 | *CRP* | 1: 159709846 | G/T | 0.079 | -0.24 | 0.58 | 0.665 | 0.080 | 0.004 | 0.007 | 0.521 | 0.062 | 0.001 | 0.003 | 0.673 |
| rs33911258 | *AVPR1B* | 1: 206118034 | G/A | 0.167 | 0.01 | 0.42 | 0.990 | 0.147 | -0.001 | 0.005 | 0.920 | 0.148 | -0.003 | 0.003 | 0.321 |
| rs1800587 | *IL1A* | 2: 112785383 | A/G | 0.278 | 0.29 | 0.35 | 0.397 | 0.296 | -0.002 | 0.004 | 0.608 | 0.287 | -0.002 | 0.002 | 0.406 |
| rs16944 | *IL1B* | 2: 112837290 | A/G | 0.378 | -0.26 | 0.32 | 0.411 | 0.358 | 0.007 | 0.004 | 0.056 | 0.333 | 0.005 | 0.002 | **0.045** |
| rs7632287 | *OXTR* | 3: 8749760 | A/G | 0.199 | -0.16 | 0.38 | 0.661 | 0.223 | 0.001 | 0.004 | 0.742 | 0.230 | 0.001 | 0.002 | 0.727 |
| rs2254298 | *OXTR* | 3: 8760542 | A/G | 0.095 | -0.13 | 0.54 | 0.795 | 0.117 | 0.002 | 0.005 | 0.734 | 0.102 | 0.001 | 0.003 | 0.682 |
| rs53576 | *OXTR* | 3: 8762685 | A/G | 0.473 | -0.04 | 0.31 | 0.874 | 0.337 | -0.000 | 0.004 | 0.980 | 0.328 | 0.001 | 0.002 | 0.811 |
| rs2228485 | *OXTR* | 3: 8768017 | G/A | 0.208 | -0.25 | 0.38 | 0.493 | 0.267 | -0.002 | 0.004 | 0.584 | 0.248 | -0.002 | 0.002 | 0.443 |
| rs237911* | *OXTR* | 3: 8768322 | G/A | 0.165 | -0.04 | 0.42 | 0.928 | - | - | - | - | - | - | - | - |
| rs9818870 | *MRAS* | 3: 138403280 | T/C | 0.139 | 0.36 | 0.46 | 0.424 | 0.162 | -0.000 | 0.005 | 0.943 | 0.163 | -0.001 | 0.003 | 0.657 |
| rs1317082 | *TERC* | 3: 169779797 | G/A | 0.341 | -0.01 | 0.33 | 0.987 | 0.250 | 0.000 | 0.004 | 0.946 | 0.245 | 0.001 | 0.003 | 0.679 |
| rs7726159 | *TERT* | 5: 1282204 | A/C | 0.337 | -0.31 | 0.33 | 0.339 | 0.337 | 0.001 | 0.004 | 0.807 | 0.352 | 0.000 | 0.003 | 0.929 |
| rs41423247 | *NR3C1* | 5: 143399010 | C/G | 0.352 | -0.04 | 0.33 | 0.898 | 0.375 | -0.006 | 0.004 | 0.084 | 0.359 | -0.003 | 0.002 | 0.232 |
| rs1800629 | *TNF* | 6: 31575254 | A/G | 0.109 | 0.04 | 0.51 | 0.931 | 0.134 | -0.003 | 0.005 | 0.545 | 0.151 | -0.003 | 0.003 | 0.274 |
| rs1041981* | *TNF* | 6: 31573007 | A/C | 0.248 | -0.46 | 0.35 | 0.193 | - | - | - | - | - | - | - | - |
| rs3800373 | *FKBP5* | 6: 35574699 | C/A | 0.234 | -0.01 | 0.36 | 0.978 | 0.302 | 0.001 | 0.004 | 0.782 | 0.306 | 0.001 | 0.002 | 0.664 |
| rs1360780* | *FKBP5* | 6: 35639794 | T/C | 0.280 | -0.11 | 0.35 | 0.752 | 0.322 | 0.000 | 0.004 | 0.936 | 0.320 | 0.001 | 0.002 | 0.736 |
| rs13212041 | *HTR1B* | 6: 77461407 | C/T | 0.177 | 0.12 | 0.40 | 0.754 | 0.192 | 0.012 | 0.004 | **0.008** | 0.214 | 0.007 | 0.003 | **0.007** |
| rs10457441 | *MIR2113* | 6: 98124244 | C/T | 0.419 | 0.48 | 0.32 | 0.128 | 0.526 | -0.011 | 0.003 | **0.001** | 0.520 | -0.007 | 0.002 | **0.003** |
| rs2148710 | *FYN* | 6: 111801023 | T/C | 0.135 | 0.32 | 0.45 | 0.480 | 0.162 | 0.008 | 0.005 | 0.086 | 0.151 | 0.004 | 0.003 | 0.141 |
| rs2715157 | *PCLO* | 7: 82839058 | A/G | 0.438 | 0.67 | 0.31 | **0.031** | - | - | - | - | - | - | - | - |
| rs531564 | *MIR124* | 8: 3445535 | C/G | 0.151 | -0.22 | 0.44 | 0.610 | 0.100 | 0.003 | 0.006 | 0.588 | 0.116 | 0.001 | 0.003 | 0.626 |
| rs2487999 | *OBFC1* | 10: 103900068 | T/C | 0.087 | 0.65 | 0.56 | 0.240 | 0.097 | 0.004 | 0.006 | 0.473 | 0.095 | 0.003 | 0.002 | 0.270 |
| rs1800955 | *DRD4* | 11: 636784 | C/T | 0.402 | -0.26 | 0.31 | 0.390 | 0.379 | 0.004 | 0.004 | 0.324 | 0.426 | 0.002 | 0.003 | 0.457 |
| rs187238 | *IL18* | 11: 112164265 | G/C | 0.283 | -0.73 | 0.35 | **0.034** | 0.269 | 0.005 | 0.004 | 0.222 | 0.279 | 0.001 | 0.002 | 0.545 |
| rs3803107 | *AVPR1A* | 12: 63147054 | T/C | 0.176 | 0.64 | 0.41 | 0.120 | 0.141 | -0.003 | 0.005 | 0.588 | 0.138 | -0.002 | 0.002 | 0.513 |
| rs1042615 | *AVPR1A* | 12: 63150429 | A/G | 0.403 | -0.03 | 0.31 | 0.920 | 0.442 | -0.004 | 0.004 | 0.285 | 0.454 | -0.002 | 0.003 | 0.348 |
| rs10459194 | *MIR135* | 12: 99039512 | C/T | 0.302 | 0.52 | 0.34 | 0.125 | 0.366 | -0.007 | 0.004 | **0.059** | 0.358 | -0.005 | 0.002 | **0.065** |
| rs2230912 | *P2RX7* | 12: 121184393 | G/A | 0.170 | -0.01 | 0.41 | 0.995 | 0.131 | -0.005 | 0.005 | 0.307 | 0.148 | -0.001 | 0.002 | 0.717 |
| rs1042173 | *SLC6A4* | 17: 30197993 | T/G | 0.454 | 0.32 | 0.32 | 0.303 | 0.533 | -0.003 | 0.003 | 0.354 | 0.597 | -0.002 | 0.002 | 0.429 |

Abbreviations: EA/NEA – effect (minor) allele/non-effect (major) allele; EAF – effect allele frequency; β – regression coefficient. A trend for statistically significant allele effects is shown in bold. Sex, ethnicity and age are included in linear regression models as covariates. Proxy SNPs in the *OXTR, TNF*, and *FKBP5* genes, which have been excluded from the PGS calculation, are marked with an asterisk. Dashes stand for SNPs, which have not been genotyped in GWAS of Neuroticism.

**Table S3.** Linear regression models demonstrating the effect of weighted and unweighted PGS and social/lifestyle factors on BDI-measured Depression in DeprVUR and women group (for sensitivity purposes).

| **№** | **Parameter** | **Total weighted**  **N=1,065** | | | **Total unweighted**  **N=1,065** | | | **Women**  **N=841** | | |
| --- | --- | --- | --- | --- | --- | --- | --- | --- | --- | --- |
|  |  | **β** | **SE** | **p-value** | **β** | **SE** | **p-value** | **β** | **SE** | **p-value** |
| **Model 1** | PGS | 57.65 | 11.23 | 3.42x10^-7^ | 13.93 | 3.68 | 1.16x10^-4^ | 68.00 | 11.57 | 6.03x10^-9^ |
|  | Model P-value | 3.42x10^-7^ | | | 1.61x10^-4^ | | | 6.03x10^-9^ | | |
|  | Adjusted r^2^ | 0.024 | | | 0.013 | | | 0.039 | | |
| **Model 2** | Sex | 1.12 | 0.54 | 0.038 | 1.12 | 0.54 | 0.038 | - | - | - |
|  | Ethnicity (Russians) | -1.34 | 0.76 | 0.079 | -1.34 | 0.76 | 0.079 | -1.04 | 0.84 | 0.21 |
|  | Ethnicity (Tatars) | -2.77 | 0.76 | 2.96x10^-4^ | -2.77 | 0.76 | 2.96x10^-4^ | -2.43 | 0.84 | 0.0030 |
|  | Ethnicity (Udmurts) | -1.95 | 0.83 | 0.019 | -1.95 | 0.83 | 0.019 | -1.34 | 0.92 | 0.14 |
|  | Age | -0.30 | 0.13 | 0.022 | -0.30 | 0.13 | 0.022 | -0.35 | 0.15 | 0.018 |
|  | Model P-value | 1.21x10^-5^ | | | 1.21x10^-5^ | | | 3.79x10^-4^ | | |
|  | Adjusted r^2^ | 0.025 | | | 0.025 | | | 0.020 | | |
| **Model 3** | PGS | 53.44 | 11.19 | 2.05x10^-6^ | 12.83 | 3.69 | 5.34x10^-4^ | 68.10 | 11.46 | 4.15x10^-9^ |
|  | Sex | 1.07 | 0.54 | 0.045 | 1.07 | 0.54 | 0.045 | - | - | - |
|  | Ethnicity (Russians) | -1.60 | 0.76 | 0.034 | -1.65 | 0.77 | 0.030 | -1.20 | 0.82 | 0.14 |
|  | Ethnicity (Tatars) | -2.74 | 0.76 | 2.95x10^-4^ | -2.82 | 0.76 | 2.09x10^-4^ | -2.41 | 0.82 | 0.0030 |
|  | Ethnicity (Udmurts) | -1.89 | 0.83 | 0.021 | -1.95 | 0.83 | 0.018 | -1.51 | 0.90 | 0.093 |
|  | Age | -0.30 | 0.13 | 0.018 | -0.29 | 0.13 | 0.022 | -0.35 | 0.15 | 0.016 |
|  | Model P-value | 1.15x10^-9^ | | | 1.49x10^-7^ | | | 1.17x10^-10^ | | |
|  | Adjusted r^2^ | 0.046 | | | 0.036 | | | 0.061 | | |
| **Model 4** | PGS | 53.60 | 13.28 | 6.1x10^-5^ | 12.96 | 4.39 | 0.0030 | 64.82 | 14.15 | 5.76x10^-6^ |
|  | Sex | 1.48 | 0.63 | 0.019 | 1.46 | 0.64 | 0.022 | - | - | - |
|  | Ethnicity (Russians) | -1.59 | 0.88 | 0.069 | -1.71 | 0.88 | 0.054 | -1.69 | 0.96 | 0.076 |
|  | Ethnicity (Tatars) | -2.51 | 0.84 | 0.0030 | -2.60 | 0.85 | 0.0020 | -2.70 | 0.92 | 0.0030 |
|  | Ethnicity (Udmurts) | -1.15 | 1.01 | 0.25 | -1.37 | 1.02 | 0.18 | -1.95 | 1.16 | 0.091 |
|  | Age | -0.27 | 0.14 | 0.060 | -0.26 | 0.14 | 0.067 | -0.32 | 0.17 | 0.054 |
|  | Income level (average) | -0.95 | 0.67 | 0.159 | -1.91 | 0.88 | 0.029 | -3.18 | 1.04 | 0.002 |
|  | Maternal care | -2.65 | 0.59 | 1.01x10^-5^ | -2.71 | 0.59 | 6.55x10^-6^ | -1.96 | 0.68 | 0.004 |
|  | Maternal protection | 1.54 | 0.56 | 0.0060 | 1.48 | 0.56 | 0.0070 | 1.33 | 0.64 | 0.038 |
|  | Paternal care | -1.13 | 0.54 | 0.036 | -1.15 | 0.54 | 0.033 | -1.51 | 0.61 | 0.013 |
|  | Paternal protection | 1.46 | 0.55 | 0.0080 | 1.43 | 0.55 | 0.0090 | 1.82 | 0.63 | 0.003 |
|  | Model P-value | <2.2x10^-16^ | | | <2.2x10^-16^ | | | <2.2x10^-16^ | | |
|  | Adjusted r^2^ | 0.15 | | | 0.14 | | | 0.17 | | |

Dashes stand for non-included items in the regression model. The best social-demographic predictors according to stepwise backward elimination procedure performed in the total sample are included in the Model 4.

**Table S4.** Sensitivity models demonstrating the effect of PGS and social/lifestyle factors based on published neuroticism GWAS in Europeans on BDI-measured Depression in DeprVUR sample.

| **№** | **Parameter** | **Okbay et al., 2016**  **weighted** | | | **Okbay et al., 2016**  **unweighted** | | | **Turley et al., 2018**  **weighted** | | | **Turley et al., 2018**  **unweighted** | | |
| --- | --- | --- | --- | --- | --- | --- | --- | --- | --- | --- | --- | --- | --- |
|  |  | **β** | **SE** | **p-value** | **β** | **SE** | **p-value** | **β** | **SE** | **p-value** | **β** | **SE** | **p-value** |
| **Model 1** | PGS | -1551.44 | 792.22 | 0.0505 | -10.06 | 4.31 | 0.019 | -2254.04 | 1254.06 | 0.073 | -7.55 | 3.73 | 0.043 |
|  | Model P-value | 0.051 | | | 0.019 | | | 0.072 | | | 0.043 | | |
|  | Adjusted r^2^ | 0.0027 | | | 0.0043 | | | 0.0021 | | | 0.0029 | | |
| **Model 2** | PGS | -1668.16 | 790.59 | 0.035 | -10.50 | 4.30 | 0.014 | -2418.03 | 1251.39 | 0.053 | -7.81 | 3.73 | 0.036 |
|  | Sex | 1.13 | 0.54 | 0.036 | 1.12 | 0.54 | 0.037 | 1.13 | 0.54 | 0.036 | 1.09 | 0.54 | 0.044 |
|  | Ethnicity (Russians) | -1.44 | 0.77 | 0.058 | -1.50 | 0.77 | 0.049 | -1.46 | 0.77 | 0.055 | -1.52 | 0.77 | 0.047 |
|  | Ethnicity (Tatars) | -2.76 | 0.76 | 3.08x10^-4^ | -2.75 | 0.76 | 3.08x10^-4^ | -2.76 | 0.76 | 2.96x10^-4^ | -2.79 | 0.76 | 2.57x10^-4^ |
|  | Ethnicity (Udmurts) | -1.98 | 0.83 | 0.017 | -2.00 | 0.83 | 0.016 | -1.97 | 0.83 | 0.018 | -2.01 | 0.83 | 0.015 |
|  | Age | -0.31 | 0.13 | 0.015 | -0.31 | 0.13 | 0.016 | -0.31 | 0.13 | 0.017 | -0.30 | 0.13 | 0.019 |
|  | Model P-value | 4.55x10^-6^ | | | 2.30x10^-6^ | | | 6.26x10^-6^ | | | 4.69x10^-6^ | | |
|  | Adjusted r^2^ | 0.028 | | | 0.030 | | | 0.027 | | | 0.028 | | |
| **Model 3** | PGS | -1485.28 | 917.25 | 0.11 | -13.09 | 5.09 | 0.010 | -2238.38 | 1470.65 | 0.13 | -9.27 | 4.44 | 0.037 |
|  | Sex | 1.85 | 0.64 | 0.0043 | 1.87 | 0.65 | 0.0042 | 1.85 | 0.65 | 0.0046 | 1.82 | 0.65 | 0.0054 |
|  | Ethnicity (Russians) | -1.70 | 0.89 | 0.054 | -1.91 | 0.88 | 0.031 | -1.77 | 0.89 | 0.046 | -1.88 | 0.89 | 0.033 |
|  | Ethnicity (Tatars) | -2.47 | 0.85 | 0.0036 | -2.44 | 0.84 | 0.0037 | -2.42 | 0.84 | 0.0041 | -2.46 | 0.84 | 0.0035 |
|  | Ethnicity (Udmurts) | -1.53 | 0.02 | 0.13 | -1.86 | 1.05 | 0.074 | -1.79 | 1.05 | 0.088 | -1.84 | 1.05 | 0.079 |
|  | Age | -0.30 | 0.14 | 0.037 | -0.36 | 0.14 | 0.011 | -0.35 | 0.14 | 0.015 | -0.35 | 0.14 | 0.015 |
|  | Income level (average) | -2.15 | 0.88 | 0.014 | -2.40 | 0.90 | 0.0075 | -2.41 | 0.90 | 0.0076 | -2.39 | 0.90 | 0.0079 |
|  | Maternal care | -2.89 | 0.60 | 1.64x10^-6^ | -2.58 | 0.60 | 2.16x10^-5^ | -2.60 | 0.61 | 2.06x10^-5^ | -2.60 | 0.60 | 1.95x10^-5^ |
|  | Maternal protection | 1.35 | 0.56 | 0.015 | 1.37 | 0.56 | 0.014 | 1.44 | 0.56 | 0.010 | 1.43 | 0.56 | 0.011 |
|  | Paternal care | -1.16 | 0.54 | 0.031 | -1.14 | 0.55 | 0.038 | -1.14 | 0.55 | 0.038 | -1.14 | 0.55 | 0.037 |
|  | Paternal protection | 1.45 | 0.55 | 0.0084 | 1.58 | 0.56 | 0.0045 | 1.50 | 0.56 | 0.0071 | 1.52 | 0.56 | 0.0062 |
|  | Model P-value | < 2.2x10^-16^ | | | < 2.2x10^-16^ | | | < 2.2x10^-16^ | | | < 2.2x10^-16^ | | |
|  | Adjusted r^2^ | 0.14 | | | 0.15 | | | 0.15 | | | 0.15 | | |

Dashes stand for non-included items in the regression model. The best predictors according to stepwise backward elimination procedure are included in the Model 4.

Supplementary Figures


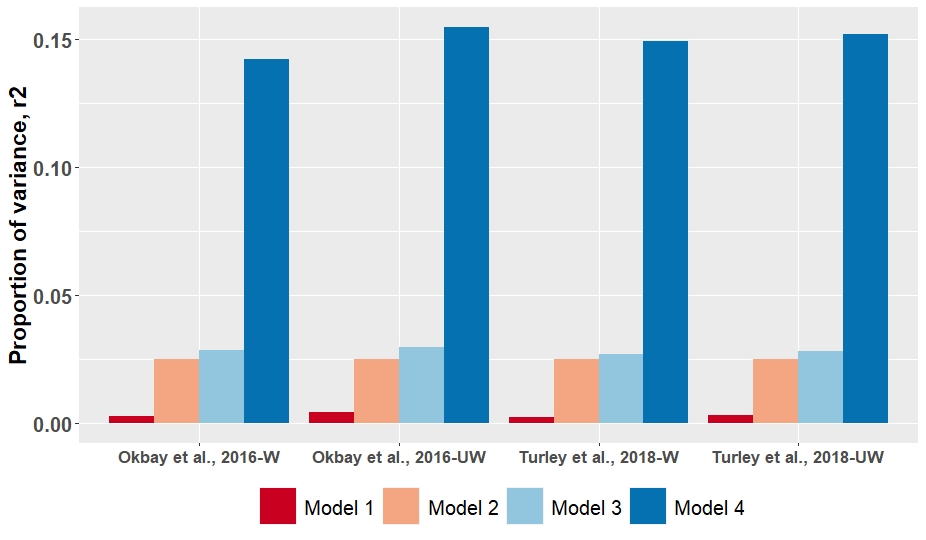


**Figure S1.** Proportion of variance (adjusted r^2^) in BDI-measured depression explained by predictors in DeprVUR sample based on weighted (W) and unweighted (UW) PGS. PGS score was calculated based on summary statistics from GWAS data on neuroticism [13,14] in Europeans. For sensitivity purposes unweighted (UW) PGS effects in the total DeprVUR sample and weighted PGS in women were examined. In all groups examined predictors in four different linear regression models were: (1) PGS; (2) sex, ethnicity, and age; (3) PGS, sex, age, and ethnicity; (4) PGS, age, sex, ethnicity, and the most significant social-demographic predictors. Included predictors are described in details in Table S4.
